# Treatment of active lupus nephritis with the novel immunosuppressant 15-deoxyspergualin: an open-label dose escalation study

**DOI:** 10.1186/ar3268

**Published:** 2011-03-01

**Authors:** Hanns-Martin Lorenz, Wilhelm H Schmitt, Vladimir Tesar, Ulf Müller-Ladner, Ingo Tarner, Ingeborg A Hauser, Falk Hiepe, Tobias Alexander, Heike Woehling, Kyuichi Nemoto, Peter A Heinzel

**Affiliations:** 1 Division of Rheumatology, University Hospital Heidelberg, INF410, 69120 Heidelberg, Germany; 2 Department of Nephrology, University Hospital Mannheim, Theodor-Kutzer-Ufer 1-3, 68167 Mannheim, Germany; 3 Department of Nephrology, 1st Faculty of Medicine, Charles University and General University Hospital, U nemocnice 2, 128 08 Prague 2, Czech Republic; 4 Justus-Liebig University Giessen, Department of Rheumatology and Clinical Immunology, Kerckhoff-Clinic, Benekestr. 2-8, 61231 Bad Nauheim, Germany; 5 Department of Nephrology, University Hospital Frankfurt/Main, Theodor-Stern-Kai 7, 60590 Frankfurt/Main, Germany; 6 Department of Rheumatology, Charité, University Hospital Berlin, Charitéplatz 1, 10117 Berlin, Germany; 7 Dabio GmbH, Ahornstr. 1, 85635 Höhenkirchen-Siegertsbrunn, Germany; 8 Euro Nippon Kayaku, Staufenstr. 4, 60323 Frankfurt, Germany

## Abstract

**Introduction:**

As the immunosuppressive potency of 15-deoxyspergualin (DSG) has been shown in the therapy of renal transplant rejection and Wegener's granulomatosis, the intention of this study was to evaluate the safety of DSG in the therapy of lupus nephritis (LN).

**Methods:**

Patients with histologically proven active LN after prior treatment with at least one immunosuppressant were treated with 0.5 mg/kg normal body weight/day DSG, injected subcutaneously for 14 days, followed by a break of one week. These cycles were repeated to a maximum of nine times. Doses of oral corticosteroids were gradually reduced to 7.5 mg/day or lower by cycle 4. Response was measured according to a predefined decision pattern. The dose of DSG was adjusted depending on the efficacy and side effects.

**Results:**

A total of 21 patients were included in this phase-I/II study. After the first DSG injection, one patient was excluded from the study due to renal failure. Five patients dropped out due to adverse events or serious adverse events including fever, leukopenia, oral candidiasis, herpes zoster or pneumonia. Eleven out of 20 patients achieved partial (4) or complete responses (7), 8 were judged as treatment failures and 1 patient was not assessable. Twelve patients completed all nine cycles; in those patients, proteinuria decreased from 5.88 g/day to 3.37 g/day (*P *= 0.028), Selena-SLEDAI (Safety of Estrogens in Lupus Erythematosus - National Assessment - systemic lupus erythematosus disease activity index) decreased from 17.6 to 11.7. In 13 out of 20 patients, proteinuria decreased by at least 50%; in 7 patients to less than 1 g/day.

**Conclusions:**

Although the number of patients was small, we could demonstrate that DSG provides a tolerably safe treatment for LN. The improvement in proteinuria encourages larger controlled trials.

**Trial registration:**

ClinicalTrials.gov: NCT00709722

## Introduction

Systemic lupus erythematosus (SLE) is an aggressive autoimmune disease. Lupus nephritis (LN) is a major complication of SLE and a strong determinant of morbidity and mortality. Standard treatment protocols for lupus nephritis involve intravenous (IV) pulses of corticosteroids and cyclophosphamide (CYC) or mycophenolate mofetil (MMF) for induction therapy, with oral corticosteroids (OCS) and azathioprine (AZA) or mycophenolic acid as long-term maintenance treatment [1-3]. Although pulsed IV CYC is effective in improving renal survival, a significant proportion of patients demonstrate poor renal response or relapses [4,5]. The optimal therapy for such patients with CYC-resistant or relapsing LN remains unclear. Moreover, CYC is associated with a substantial side-effect profile [6]. The risk of these side effects remains higher for more than 10 years after termination of CYC treatment, and is especially high if the patients received a cumulative dosage of >36 g [7-9].

15-deoxyspergualin (DSG; Gusperimus) shows immunosuppressive activity both *in vitro *and *in vivo*, affecting B-lymphocyte, T-lymphocyte and macrophage/monocyte function. In rodents and human cell systems, DSG shows a dose-dependent inhibition of primary and secondary responses to T-, B- and antigen-presenting cell dependent reactions [10-19]. It has been demonstrated that DSG binds with high affinity to heat shock protein c (hsc) 73 [20,21]. DSG also blocks nuclear translocation of NF-κB in a pre-B-cell line, thereby affecting NF-κB-driven transcription of the kappa light chain [20-23]. Finally, Nishimura *et al. *[24] reported that DSG inhibits desoxyhypusine synthase, the first enzyme in the formation of active eukaryotic translation initiation factor 5A. This factor is important for the stabilization of certain mRNA transcripts (TNF-α and others).

The immunosuppressive properties of DSG have been demonstrated in preclinical animal studies including SLE models [25-31]. In humans with glucocorticoid-resistant kidney transplant rejection, DSG shows the same efficacy rate as the strongly T-cell depleting anti-CD3 monoclonal antibody [32,33]. DSG has been licensed in Japan for acute renal allograft rejection since 1994. In 2003, an open clinical trial successfully tested DSG in patients with persistent ANCA-associated vasculitis [34-36]. Adverse events (AE) were common but rarely led to treatment discontinuation. Against this background, DSG was granted an orphan drug status for the treatment of Wegener's granulomatosis by the European Medicines Agency (EMA).

As DSG induces a reversible maturation block of granulocytes, it needs to be administered in cycles with intermittent wash-out periods. In the previous studies, it was concluded that the degree of the clinical response does not correlate to the severity or duration of leukopenia elicited in the individual patient. This was an important influence on the protocol for our current SLE study: for safety reasons, we shortened the treatment intervals and started with lower dosages, as SLE patients are more prone to leuko- and lymphocytopenia than patients with Wegener's granulomatosis. In human studies on cancer treatment, in contrast, DSG was applied intravenously at much higher dosages and was still generally well tolerated [37,38]. The study presented here was also encouraged by beneficial results achieved when three patients with active LN were treated with DSG using the same protocol as used here [39]. All three patients had been treated with various immunosuppressives including cyclophosphamide; after informed consent, we started treating with DSG along with corticosteroids, which could be gradually reduced within the first cycles. Indicators of response were a decrease of proteinuria, hematuria and an improvement in the serological parameters of lupus activity [39].

Thus, based on the favourable toxicity profile of DSG, the limited number of immunosuppressants available for the treatment of aggressive SLE, the sometimes considerable side effects of cyclophosphamide as the best evaluated immunosuppressant for treatment of aggressive SLE, the good efficacy and safety data for DSG in the treatment of Wegener's granulomatosis, and the favourable data from the three previously mentioned patients with LN, we initiated this multicenter open phase I/II trial of DSG in the treatment of refractory LN.

## Materials and methods

### Study design

The purpose of this open-labeled, multicenter, single group, dose-finding phase I/II pilot study was to establish the dose of DSG which reduces LN activity after a minimum of six cycles of treatment without causing World Health Organization (WHO) grade 3 leukopenia (WBC <2 × 10^9^/L). This was important, as DSG causes reversible leukocytopenia, lupus patients are prone to leukocytopenia as a consequence of the disease itself, and there is limited data about the long-term treatment of SLE with DSG. We, therefore, deviated from Wegener's protocol and reduced both the initial dosage and the cycle duration with DSG. The patients, who had all been previously treated with standard immunosuppressants, suffered from persistent LN and were on OCS (≤1.0 mg/kg/day; maximum dose 80 mg/day) at entry into the trial. The study was in accordance with the ethical standards of the Helsinki Declaration. The study was registered at ClinicalTrials (Identifier: NCT00709722).

### Endpoints

The response rate as the final outcome of the study was the primary endpoint. A four-point scale was defined: complete response (CR), partial response (PR), stable disease (SD) or treatment failure (TF). The response criteria were defined prior to the start of the study (Table 1): for a CR, PR or SD prednisone had to be decreased to ≤7.5 mg/day, a higher dosage was automatically classified as TF. The presence of urinary erythrocyte or granular casts excluded CR. As the baseline activity of every patient is different (renal function, baseline proteinuria), it was necessary to define baseline proteinuria (g/24 h) or kidney function (estimated glomerular filtration rate (EGFR), according to the Cockgroft-Gault formula) as the reference value for the definition of response for every patient individually. The baseline was defined as the renal function and proteinuria level before the onset of the recent LN flare which qualified the patient for the study. Response was, therefore, determined as the ratio of the proteinuria or kidney function at cycle 4, 6 or 9 to the baseline values of the individual patient. Thus CR, PR, SD or TF could be determined according to the scheme as depicted in Table 1. Patients with CR or PR were called "responders" while those with SD or TF were "non-responders" to DSG.

**Table 1:** Definition of response criteria

		Complete response	Partial response	Stable disease	Treatment failure
Criteria	Baseline	Criteria 1 to 4 must be fulfilled	Criterion 1 must be fulfilled, and either 3 or 4, with the other not downgrading clinical response to SD or TF	Criterion 1 must be fulfilled, moreover 3 or 4, with the other not downgrading clinical response to TF	If one of criteria 1, 3 or 4 accounts
1. prednisone equivalent)		< = 7.5 mg/day	< = 7.5 mg/day	< = 7.5 mg/day	➢ 7.5 mg/day during cycle 4, 6 or 9
2. Urinary casts		Not detectable	Detectable	Detectable	Detectable
3. Proteinuria	A) Normal (< 0.15 g/day)	< 0.3 g/day	> 0.3 g, but a decrease of > = 25% of the maximum urinary protein excretion (measured at entry) achieved	decrease of <25% of the maximum urinary protein excretion (measured at entry) achieved during DSG treatment, no further increase of >25% in the maximum urinary protein excretion within the previous two cycles	Within the previous two cycles, a further increase of >25% in the maximum urinary protein excretion
	B) Elevated	Maximum increase over baseline of 25%	If >25% increased additional urinary protein excretion^1 ^was decreased by at least 25% during DSG treatment	additional urinary protein excretion^1 ^decreased by < 25% during DSG treatment; no further increase of >25% in the maximum urinary protein excretion within the previous two cycles	Within the previous two cycles, a further increase of >25% in the maximum urinary protein excretion
	C) In case of chronic nephrotic syndrome	Decrease in proteinuria of >50%, compared to the baseline	Decrease in proteinuria of at least 25%, but less than 50%, compared to the baseline	Decrease in proteinuria of <25%, maximal increase of 25%, compared to the baseline	Further increase in proteinuria of >25%, compared to the baseline
4. Serum creatinine and EGFR	A) Both normal	Serum creatinine normal and impairment of EGFR^2 ^improved by at least 75%	Serum creatinine normal and impairment of EGFR^2 ^improved by at least 25%, but less than 75%	Serum creatinine remained elevated or impairment of EGFR^2 ^improved by <25%, but did not further decrease by >25% within the previous two cycles	serum creatinine remained elevated, with a further increase of >20% over the maximum serum creatinine occurring within the previous two cycles or impairment of EGFR^2 ^further increased by >25% within the last two cycles
	B) Decreased EGFR, normal serum creatinine	Serum creatinine normal and impairment of EGFR^2 ^improved by >= 75%	Serum creatinine normal and impairment of EGFR^2 ^improved by >= 25%, but <75%	impairment of EGFR^2 ^improved by <25% or further decreased to <= 25% under the minimum EGFR within the last previous cycles	EGFR further decreased by >25% under the minimum EGFR within the previous two cycles
	C) Elevated serum creatinine	Maximum increase 20%	If >20% higher than baseline serum creatinine, at least a decrease from maximum creatinine during the trial of >15%	Serum creatinine concentration +/- 15% around the maximum value observed during the DSG trial	During the last two cycles, serum creatinine further increased by >15% over the maximum value observed during the DSG trial

Baseline was defined as proteinuria or renal function (serum creatinine and EGFR) before the current flare of LN. According to these entry parameters, each patient was attributed to group 3A, 3B or 3C and 4A, 4B or 4C, respectively. Next, the maximal proteinuria or maximal serum creatinine/minimal EGFR during the current LN flare was determined. Based on these numbers, the additional urinary protein excretion (maximal amount of proteinura - baseline proteinuria) and/or impairment of renal function (maximal creatinine - baseline creatinine; baseline EGFR - minimum EGFR) could be defined for each patient individually. Response to DSG at the end of cycle 4, 6 or 9 was then defined in relation to the patient's individual entry parameters according to the criteria in Table 1.

DSG, deoxyspergualin; EGFR, estimated glomerular filtration rate; SD, stable disease; TF, treatment failure.

^1 ^additional urinary protein excretion: maximal amount of proteinuria (g/day) - baseline proteinuria

Secondary endpoints in this study were: incidence of WHO grade 3 leucopenia and incidence of infections or other adverse events; responder/non-responder per dosage of DSG; time and duration of response and Selena-SLEDAI (Safety of Estrogens in Lupus Erythematosus - National Assessment - systemic lupus erythematosus disease activity index) score during treatment; treatment days with corticosteroids of ≤7.5 mg/day. An AE was defined as any adverse deviation from the patient's baseline condition during the trial (including laboratory abnormalities, intercurrent diseases and accidents), whether or not the change was considered to be related to the study drug. As usual, the events were categorized as mild, moderate or severe by the clinical investigator at the study center. A serious adverse event (SAE) was an event which is life-threatening, results in death, requires or prolongs hospitalization or results in persistent or significant disability/incapacity.

Reasons for discontinuing treatment with DSG were: onset of intercurrent diseases which did not allow the continuation of DSG treatment; common toxicity criteria (CTC) grade 3 suppression of WBC, neutrophils, hemoglobin, or platelets; withdrawal of consent by the patient; decision by the physician that discontinuation was in the best interest of the patient; pregnancy; life-threatening complications; increase in serum creatinine >5 mg/dL; development of cerebral lupus; and progression of the disease that did not justify the continuation of DSG therapy (for example, treatment with OCS (prednisolone equivalent) >1 mg/kg/day or treatment with CYC required). All patients with premature termination were included in the safety analysis. If the duration of treatment was at least four cycles, the efficacy of treatment was assessed, too (intention-to-treat analysis, ITT). Therapy after the patient's withdrawal from the study was left at the discretion of the investigator.

### Patients

Inclusion and exclusion criteria are listed in Table 2. Conventional immunosuppressants had to have been stopped at least one week before DSG treatment was started. Concomitant use of these immunosuppressants was excluded. Daily OCS doses of 1.0 mg/kg or less (maximum daily dose 80 mg) were allowed at the start of DSG therapy. Female patients of child-bearing age had to use safe methods of contraception. Any other condition that might have rendered the patient unsuitable for participation in the study was regarded as an exclusion criterion.

**Table 2:** Inclusions and exclusion criteria

Inclusion criteria	Exclusion criteria
Age between 18 and 70 years	Chronic infection with HIV, Hepatitis B or Hepatitis C
Diagnosis of SLE according to the ACR criteria	Acute severe infection including fungal, viral, bacterial or protozoal diseases
Signs of active SLE nephritis: increasing urinary protein excretion of 1 g or more per 24 hours (if initially normal values) or a further increase of >50% over the baseline proteinuria and/or active urinary sediment and/or impaired renal function due to SLE nephritis (newly elevated serum creatinine	Signs of liver toxicity (WHO common toxicity criteria class 2 and higher)
If initially normal values - or >50% increase of serum creatinine levels if elevated before onset of renal flare), or signs of active LN in renal biopsy (any renal biopsy in the past two years)	Absence of adequate liver function (total bilirubin >25 μmol/L = 1.4 mg/dL unless otherwise explained (for example, inherited, hemolysis), ALT or AST >2.5 times upper limit of normal values)
Serum creatinine concentration of μ5.0 mg/dL	
	Anemia (hemoglobulin <8.0 g/dL)
Prior treatment with one or more immunosuppressive drugs (for example, CYC, AZA, methotrexate, cyclosporin A, MMF), or plasmapheresis	
	Leukopenia (leukocytes <4,000/µL unless attributable to SLE: leukocytes <2,000/µL in these cases)
Initial leukocyte count >4,000 cells/µL (unless leukopenia due to SLE disease activity: leukocyte count:/2,000/µL	
	Thrombocytopenia (platelets <50,000/µL),
Written informed consent	
	Neutrophil counts below 1,000/µL
	Hypogammaglobulinemia (IgG below 400 mg/dl)
	Pregnancy or lactation
	Major and active SLE organ involvement other than the kidney, especially CNS involvement
	History of malignancy
	Participation in another clinical trial within six months before screening

^2 ^= difference of baseline EGFR minus minimum EGFR during DSG trial from entry

ACR, American College of Rheumatology; AZA, azathioprin; CYC, cyclophosphamide; LN, lupus nephritis; MMF, mycophenolic acid; SLE, systemic lupus erythematosus.

### Treatment protocol

Patients were treated for a maximum of nine treatment cycles with DSG. Treatment was started with a daily dose of 0.5 mg/kg normal body weight/day, injected s.c. for 14 days, followed by a break of one week (= one cycle). OCS dosage was maintained, decreased, or increased according to the response to DSG.

On the last day of the fourth, sixth and ninth cycle, the investigator assessed the response using the criteria specified in Table 1. After cycle 4, the daily dose of DSG in the subsequent cycles was lowered to 0.35 mg/kg/day, kept stable at 0.5 mg/kg/day or increased to 0.7 mg/kg/day, depending on response and/or toxicity. After cycle 6, the dose was again adjusted according to response and/or toxicity, to 0.25 mg/kg/day, 0.35 mg/kg/day, 0.5 mg/kg/day, 0.7 mg/kg/day or 1.0 mg/kg/day.

### Corticosteroid therapy

Entry to the study was permitted for patients with doses of OCS of ≤1.0 mg/kg/day (maximum dose 80 mg/day). To allow a response to be defined as CR, PR or SD, OCS dosage had to be gradually reduced down to ≤7.5 mg by Day 1 of cycles 4, 6 or 9. In case OCS dosages were higher than 7.5 mg/day at Day 1 of cycle 4, 6 or 9, response was judged as TF (Table 1).

### Patient characteristics

In accordance with the entry criteria, all patients in the ITT and per protocol (PP) population met at least four of the 11 ACR criteria for the classification of SLE and suffered from active LN. All patients in the ITT population were anti-nuclear antibodies (ANA) positive, most were dsDNA antibody positive. All patients included were Caucasian. Three patients were males and 17 patients were females. The mean age was 31.3 years. Table 3 shows the patients characteristics including age, time since first diagnosis of SLE, time since first diagnosis of LN, LN WHO type, pretreatment of LN within six months before study start.

**Table 3:** Patient characteristics

CRF #	Age (years)	Time since first diagnosis of SLE	Time since first diagnosis of nephritis	LN-WHO type	Pre-treatment of LN within six months before study start
9	20	1.5 years	1.5 years	IV	Prednisone, AZA, MMF, HCQ
10	34	2.5 years	2.5 years	IV	CYC, AZA, Prednisone
11	46	11 years	11 years	IV	Prednisone, MMF
13	39	8. 5 years	4 years	V	Prednisone, MMF
14	20	2 years	4 years	IV	MMF
15	37	7 years	7 years	IV	CYC, Prednisone, CSA, AZA
16	30	17 years	17 years	IV	Prednisone,
17	40	21.5 years	21.5 years	III	AZA, Prednisone, MMF, Immunadsorption
19	20	4.5 years	4.5 years	IV	AZA, MMF, Prednisone, Methylprednisolon, HCQ, Rituximab, Octagam
26	35	9 years	8 years	IV	Prednisone, MMF
31	22, 5	3 years	3 years	IV	CYC, Prednisone
32	42	12 years	12 years	V	AZA, Methylprednisolon
33	31	7 years	7 years	IV	MMF, Prednisone, HCQ
34	19	1 year	1 year	IV	AZA, CYC, Prednison
35	42	10.5 years	10.5 years	IV	Prednisone, AZA
36	30	13 years	4 years	IV	Methylprednisolon AZA
38	22	2.5 years	2.5 years	IV	CYC, Prednisone, HCQ
39	46	2 years	2 years	IV	HCQ, plasmapheresis Prednisolone
42	29	10 years	1 year	V	Prednisone, CSA
49	37, 5	4 years	4 years	V	Prednisone, CSA
50	20	1 year	1 year	IV	Prednisone, AZA, MMF, CYC, Prednisone

AZA, azathioprin; CRF, case report form; CSA, cyclosporine A; CYC, cyclophosphamide; HCQ, hydroxychloroquine; LN, lupus nephritis; MMF, mycophenolic acid; SLE, systemic lupus erythematosus.

The diagnosis of LN was confirmed in all patients included in the ITT and PP population, with a minimum duration of 1.1 year since diagnosis. The mean duration of SLE was 7.2 years and of LN 6.1 years. According to 1995 WHO classification criteria, 16 patients suffered from diffuse proliferative nephritis (type IV) while four patients had a type V (lupus membranous nephropathy); only one patient had a focal proliferative nephritis (type III). Hematuria and proteinuria was present in all patients.

Most patients had been previously treated with more than one of the standard medications for LN. The following previous immunosuppressive therapies had been applied to the patients: predniso(lo)ne (19 patients), azathioprine (10), cyclophosphamide (5), mycophenolic acid (9), cyclosporine A (3) and rituximab (1).

All patients had terminated the respective immunosuppressive therapy, with the exception of OCS, at least one week before the start of the treatment with DSG. All patients had been on these therapies at least three months before the start of the study.

All co-medication was recorded in the case report forms (CRFs). Initiation of treatment with angiotensin converting enzyme (ACE)-inhibitors or AT II receptor antagonists or non steroidal antirheumatic drugs was avoided during the trial as these drugs can improve proteinuria or increase serum creatinine levels, thereby interfering with response-defining parameters. For patients chronically treated with any of these drugs, the medication was continued at the identical dosage.

The study protocol, including all amendments, informed consent form and patient information sheet, was approved by the Ethics Committees before the start of the study. The study was performed according to the German Drug Law, the Czech Drug Law and to the revised version of the Declaration of Helsinki from 1996. Local laboratories were certified and provided the respective documentation as well as the normal ranges.

### Laboratory tests, statistical analysis

Urine sediment was evaluated in nephrological laboratories of the participating centers. Complement levels were determined turbitimetrically, dsDNA Ab titers by Farr assay. Statistical analysis was performed with paired non-parametric Wilcoxon test.

## Results

The safety population comprised all 21 patients, the ITT population 20 patients as one patient dropped out after the first injection in cycle 1, due to an increase in serum creatinine (rated as SAE). One patient was taken off the study after cycle 4, three patients after the fifth cycle, and four patients after the sixth cycle. Twelve patients were treated for all nine cycles.

DSG dose remained unchanged in one patient over nine cycles; in three patients, DSG was reduced to 0.35 mg/kg/day (all patients were excluded from the study after five to six cycles). Fifteen patients received 0.7 mg/kg/day of DSG starting at cycle 5; in five patients DSG could be increased to 1.0 mg/kg/day in cycles 7 to 9.

Intermittent leukopenia (a known side effect of DSG) of grade 3 according to WHO classification (< 1.0 to 1.9 × 10^9^/L) was observed in seven patients during the course of the trial; however, it was observed in two cycles in only one patient. Importantly, neither the severity of leukopenia nor the DSG dosage correlated to the frequency and severity of side effects.

Overall, 329 AEs were reported in the 21 patients (Table 4, 5, 6). The most frequently reported AEs were infections and infestations (59 reports in 18 patients; Table 5), followed by gastrointestinal disorders (52 reports in 16 patients) and general disorders/injection site reaction (39 reports in 17 patients). A total of 218 of 329 AEs were of mild intensity. A relationship with the administration of DSG was assessed as possible in 86 AEs (18 patients), as probable in 37 AEs (13 patients), and definite in 6 AEs (4 patients). In most of the AEs (299), the patients remained in the trial. Sixteen patients received additional therapy due to 81 AEs. Eight patients experienced 18 SAEs (Table 6), seven patients were hospitalized and five patients terminated the study due to SAEs including fever, leukopenia, oral candidiasis, herpes zoster or pneumonia with a consecutive SLE-flare (Table 6). No deaths occurred during the study and the follow-up period. Again, DSG dosage and number or severity of side effects did not correlate.

**Table 4:** Summary of AEs and their relation to DSG treatment, outcome

		Number of AE records	Number of patients
		n	n
Total AE		329	21
Type of AE	Infections and infestations	59	18
	Gastrointestinal disorders	52	16
	General disorders/administration site condition	39	17
Intensity	Mild	218	21
	Moderate	90	18
	Severe	17	7
Outcome	Resolved	221	21
	Additional therapy	81	16
	Hospitalization	14	7
	Premature termination	8	5
Relationship to DSG	No	156	20
	Unlikely	42	15
	Possibly	86	18
	Probably	37	13
	Definitely	6	4

AE, adverse event; DSG, deoxyspergualin.

**Table 5:** Listing of infections and infestations

Infections and infestations	Number of AE	**Number of pat**.
Urinary tract infection	12	6
Oral candidiasis	7	6
Vaginal candidiasis	5	4
Nasopharyngitis	5	4
Respiratory tract infection	4	3
Bronchitis	4	2
Pneumonia	3	3
Herpes simplex	3	2
Herpes zoster	3	2
Dental caries	1	1
Fungal skin infection	1	1
Gasteroenteritis	1	1
Infected insect bite	1	1
Labyrinthitis	1	1
Onychomycosis	1	1
Otitis media	1	1
Pharyngitis	1	1
Rhinitis	1	1
Sialoadentitis	1	1
Tinea infection	1	1
Tonsillitis	1	1
Tooth infection	1	1

AE, adverse event

**Table 6:** Overview of the SAEs during the study or the post-study observation period

Patient	Description of the event	No DSG cycles	Intensity	Relationship to DSG	Action taken
A	Renal failure (Severe proteinuria)	9	Moderate	No (cycle 9, incompliance)	Hospitalization Study termination
	Parodontitis, tooth infection, fever		Moderate	No	
B	Oral candidiasis	6	Moderate	Probably	Hospitalization
	Fever		Moderate	Probably	Hospitalization
	Fever		Mild	Possibly	Study termination
	myalgia		Mild	Unlikely (during follow-up)	
	Headache		Mild	Unlikely (during follow-up)	Hospitalization
C	Angina pectoris	4	Moderate	No	Hospitalization
	Pneumonia		Severe	Probably	Hospitalization Study termination
D	Increase in serum creatinine (renal failure)	0	Severe	No (drop-out after first dose in cycle 1)	Additional therapy Study termination
E	Excision of an uterine myoma	9	Not applicable	No (during follow-up)	Hospitalization
F	Leukopenia (two SAEs)	5	Severe	Possibly	Hospitalization Study termination
	Increased lupus activity with increased proteinuria and pain		Severe	No (during follow-up)	Hospitalization
	Cyclophosphamide induced leukopenia		Severe	No (during follow-up)	Hospitalization
	Hospitalization for a second cyclophosphamide pulse		Not applicable	No (during follow-up)	Hospitalization
G	Herpes zoster	9	Moderate	Possibly	Hospitalization Study termination
H	Lupus flare (arthritis, myalgia, skin rash)	9	Moderate	Unlikely	Hospitalization

DSG, deoxyspergualin; SAE, serious adverse event.

**Figure 1:**
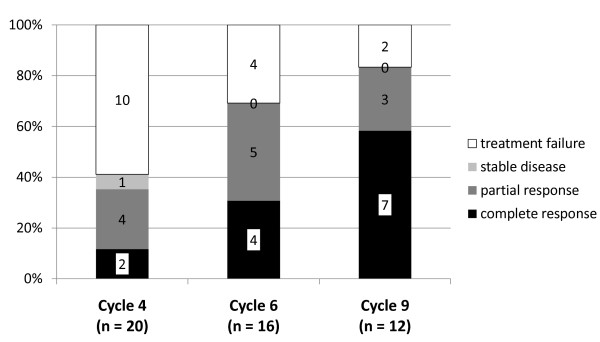
**Response rate during DSG treatment**. Response rate (CR in black, PR in dark grey, SD in bright grey, TF in white) at cycles (CYC) 4, 6 and 9 (ITT population). *In both cycles 4 and 6, three patients were not assessable.

Based on the predefined response criteria, 11 of 20 (55%) patients achieved PR (four) or CR (seven) on their final visits (one patient with PR after cycle 5, all other patients after cycle 9), eight (40%) were judged as TF on their final visit. Importantly, of these eight patients, two first responded well and achieved PR or CR, but consequently experienced a flare of their LN. Both patients had incompliantly stopped application of DSG, thus they had to be rated as TF. In one patient, response was not assessable due to missing data. Figure 1 shows the responses at cycles 4, 6 and 9. Sixteen patients completed cycle 6 (four in CR, five in PR, four as TF; for three patients, data were missing for the definition of response). Twelve patients were treated for the full nine cycles, with seven patients finishing DSG therapy in CR, three in PR. Six patients never improved, however, 14 of 20 (70%) patients improved to at least PR at some point during the study.

Proteinuria decreased significantly: at screening, the patients in the study population had a mean protein excretion of 5.124+/-4,379 g/day (range 0.248 to 20.880; *n *= 20; missing entry data on proteinuria for one patient), this decreased to 3.374+/-4,787 g/day in those 12 patients who were treated for all nine cycles. Table 7 summarizes the average proteinuria at entry and at cycles 4, 6 and 9 for the overall study population. In the 12 patients who were treated through all cycles, proteinuria fell from 5.883+/-5,503 g/day to 3.374+/-4,787 g/day (*P *= 0.028). The increase from cycle 6 to cycle 9 is mainly due to a 6- to 10-fold increase in proteinuria in the two patients who had incompliantly stopped application of DSG (patients with CRF 10 and 31 in Table 8). In 13 of 20 patients, proteinuria decreased by 50% (Table 8); in 7 patients, to less than 1 g/day (levels on entry: 1.13 to 20.88 g/day); and in 9 patients, proteinuria fell below the baseline values before onset of the recent LN flare. Only one of four patients with WHO type V LN responded (partially) to DSG; the patient with WHO type III did not improve.

**Table 7:** Proteinuria during DSG treatment: proteinuria (g/day) in the study population (*n *= patient number)

	Patient number (n)	Proteinuria (study population)	*P*-value compared to entry
Entry	20	5.124 +/- 4,379	
cycle 4	20	2.604 +/- 2,580	0.0045
cycle 6	14	2.603 +/- 2,521	0.0392
cycle 9	12	3.374 +/- 4,787	0.028

DSG, deoxyspergualin

**Table 8:** Proteinuria over the study period in patients with a 50% decrease of proteinuria (mg/day)

Patient CRF number	Baseline	Entry	Cycle 4	Cycle 6	Cycle 9
9	2,100	6,800	1,800	n.a.	1,782
10	3,600	20,880	10,710	1,572	15,576
13	2,500	3,022	1,073	2,793	
16	360	1,130	1,240	300	230
19	1,000	2,200	1,700	n.a.	1,000
26	120	5,180	800	700	
31	1,900	3,920	3,200	1,800	10,800
34	300	3,800	900	1,360	270
35	630	2,000	240	160	480
36	1,680	1,700	770	700	340
39	1,976	n.a.	2,274	2,331	1,058
49	1,700	4,600	2,800	2,900	2,360
50	5,000	11,200	750	2,770	6,401

CRF, case report form.

The analysis of urinary erythrocyte and granular casts revealed casts at screening and study entry for eight patients. In all but one patient, casts disappeared at the latest by cycle 9. At screening, patients of the ITT population had a mean EGFR of 83.75 ml/minute (range 34 to 179 ml/minute). By the end of cycle 2, mean EGFR increased to 91.57 ml/minute. During the subsequent treatment cycles, EGFR was generally stable with mean values ranging between 88.45 ml/minute (cycle 5) and 107.81 ml/minute (cycle 9). Due to the high variability and the low number of patients in this trial this did not reach statistical significance.

Interestingly, SLE-associated rashes improved in six out of eight of the affected patients (completely in four patients, partially in two). Selena-SLEDAI scores were calculated at entry, on the last day of cycles 4, 6 and 9 and at each follow-up visit. The overall scores decreased from a mean of 16.9 (12 to 32; *n *= 20) at screening to 12.9 (4 to 21; *n *= 20), 13.7 (4 to 22; *n *= 15) and 11.7 (6 to 21; *n *= 12) at the end of cycles 4, 6 or 9, respectively (again, due to the high variability and the low number of patients in this trial this did not reach statistical significance). In the 12 patients who were treated through all nine cycles, Selena-SLEDAI score decreased from 17.6 at entry to 11.7 at the end of cycle 9. The most frequent parameters scoring for the Selena-SLEDAI at the end of the study were low complements, positive dsDNA Ab titers, pyuria, hematuria, rash and arthritis, The response was maintained: at follow-up visits 1, 2 and 3, the average scores were 11.7 (*n *= 16), 12.2 (15) and 12.0 (13), respectively.

**Figure 2:**
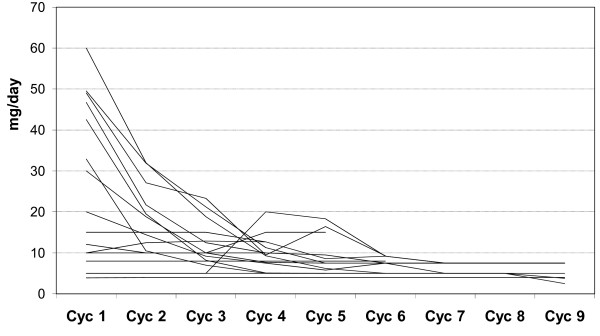
**Daily OCS dosage over DSG cycles**. Each line represents one patient.

Steroid dosage, an indirect measure of treatment efficacy, could be decreased throughout the cycles as shown in Figure 2. The number of days on which the predniso(lo)ne dose was lower than 7.5 mg/day increased continuously with treatment cycle, from an average of 2.8 days during cycle 1 to 18 days during cycle 9. Complement C3 (screening: 0.70 +/- 0.23 g/L, cycle 9: 0.76 +/- 0.25 g/L) and C4 (screening: 0.08 +/- 0.05 g/L, cycle 9: 0.15 +/- 0.20 g/L) concentrations tended to increase, C-reactive protein (CRP) (screening: 4.59 +/- 6.88 g/L; cycle 9: 2.58 +/- 3.21 g/L) and dsDNA antibody levels decreased (screening: 287.6 +/- 277 U/ml, cycle 9: 160.15 +/- 134 U/ml) (*P *> 0.05 for CRP and dsDNA Ab titers).

In the follow-up period after DSG therapy, two patients (both TF during DSG therapy) received CYC and four patients received MMF (two patients with CR during DSG therapy for maintenance, two patients with TF despite DSG therapy for induction therapy); data on three of those patients are available and indicate stable disease. Five patients were treated with rituximab (1 CR, 1 PR, 3 TF during DSG therapy); one of those patients still flared and three patients experienced a complete response. Five patients were treated with AZA and another with Cyclosporine A (CSA) (for maintenance therapy; all PR or CR with DSG therapy).

## Discussion

In this trial, we investigated the safety of DSG in therapy of lupus nephritis. The trial was encouraged by the beneficial effects observed in three patients with lupus nephritis who had been treated previously with DSG [39]. Overall, we included 21 patients; one patient was excluded after the first injection due to non-drug-related adverse events. For the ITT population, 20 patients were evaluable. Furthermore, we chose a regimen which would facilitate the identification of the appropriate DSG dosage in SLE. This was especially important as DSG induces intermittent leucocytopenia, and lupus patients are prone to leucocytopenia.

Only one patient had decreased leukocyte counts when entering the study. During treatment with DSG, this low leukocyte count did not decrease further under the expected limits during DSG treatment. As expected by the known side effects of DSG, 13 of the 21 patients suffered from leucocytopenia at at least one point during the treatment period. As in DSG trials in patients with Wegener's granulomatosis, the incidence of infections did not correlate to the degree of leukopenia.

Overall, treatment with DSG as proposed in the study protocol seems to be reasonably safe. The drop-out rate is partially explained by the early phase of clinical development of DSG, in which one needs to be cautious and withdraw patients early if there is uncertainty about the causes of AEs. As seen with other immunosuppressants, an increased rate of infections needs to be envisioned. However, it is important to remember that most of the patients had received other potent immunosuppressants in their disease history, and it is known that such patients are particularly prone to infections [40]. The treatment duration, a maximum of 27 weeks, is too short to estimate the long-term effects of DSG. Thus, safety must be considered in the future trials with DSG. With this proviso, however, DSG seems to be reasonably well tolerated.

Another aim of the study was to get an idea of the required dosage of DSG in the treatment of LN. The protocol involved treating patients with 0.5 mg/kg/d s.c. for two weeks, followed by a seven-day break to give the bone marrow time to compensate for the DSG-related intermittent leukocyte maturation block. This was an adaptation of the protocol for the treatment of Wegener's granulomatosis, in which DSG was injected daily until leucocyte counts dropped below 4,000 cells/μl. As SLE patients are prone to leukopenia *per se*, we decreased the starting dosage of DSG to 0.5 mg/kg/d and limited the injection period of DSG to 14 days. Thus, therapy was easy to handle without the frequently required blood count controls. This protocol might, therefore, offer advantages over the 'Wegener protocol', at least for the initial cycles. In terms of efficacy; however, the initial dosage of 0.5 mg/kg/d might have been too low, as for 16 of 20 patients who tolerated the drug, the dosage subsequently had to be increased to at least 0.7 mg/kg/d. Therefore, in further trials, we recommend either starting with higher dosages or increasing the dosage to a 0.7 or 1.0 mg/kg/d (or even higher if required and tolerated) faster and earlier than after cycle 4 (as in this protocol). We aimed to treat patients for a maximum of nine cycles (two weeks on drug, one week off drug = one cycle). Thus, in the best scenario in this study, patients were treated with DSG (+ low-dose glucocorticoids) for a maximum of 27 weeks. Of the 21 patients, we excluded one patient after the first injection. Of the remaining 20 patients, 12 were indeed treated for 27 weeks according to the protocol, with 5 patients reaching the maximal dosage of 1.0 mg/kg/d.

Efficacy was defined according to the response criteria detailed above and in Table 1. This method of determining the response in LN allows the improvement to be assessed individually, as patients differ in their baseline settings. Based on these parameters, only 4 of the 16 patients completing at least cycle 6 were defined as TF and taken off the study. Of the 12 patients reaching cycle 9, 7 finished as CR, 3 as PR, and 2 further patients reached CR or PR after cycle 6, then experienced a flare with increasing proteinuria due to incompliance in cycles 7 to 9 (rated as TF; Table 7). Of course, we cannot attribute this therapeutic response to DSG alone, as all patients started with elevated dosages of corticosteroids along with DSG. However, 10 out of 21 patients had been unsuccessfully treated with at least 20 mg/day of corticosteroids before the start of DSG during this SLE flare. Nonetheless, the low number of patients and the lack of a control group with an alternative treatment strategy prohibit any definite conclusion to be drawn from this trial on the efficacy of DSG in the treatment of LN.

Only a controlled randomized trial can help to define the efficacy of DSG in therapy of LN.

During treatment, renal function was stable in all patients, despite active LN at inclusion. Interestingly, in one patient, creatinine concentrations normalized from values of 1.8 mg/dl at the end of cycle 1, remained normal throughout the study and again increased during follow-up. In three patients, renal function was impaired after termination of DSG-treatment despite treatment with MMF or CYC, AZA and immunoglobulins, respectively.

Most patients suffered from WHO type IV nephritis; only four patients had WHO type V glomerulonephritis (GN). Proteinuria is one of the best predictors for end stage renal failure [41]. In 13 patients of the ITT population, proteinuria decreased by at least 50% (all WHO type IV GN), indicating that DSG (in combination with OCS) seems to affect protein excretion. Remarkably, in nine patients, proteinuria fell below the baseline values from before the onset of the recent LN flare. Amongst patients with WHO type V GN, DSG improved proteinuria in one patient only.

The Selena-SLEDAI index as a composite SLE activity score decreased by four to five points during the trial in the overall study population, and by almost six points in the 12 patients who were in the study for the full nine cycles. This compares to a decrease of 7 points (starting from the lower level of 12.1) after CYC/rituximab combination therapy [42] or a decrease of 3.6 points after Rituximab monotherapy [43] in other SLE-studies.

## Conclusions

Treatment of LN with DSG (in combination with OCS) appears to be reasonably safe with tolerable side effects, but based on the experience with the patients in this study, the dosing regimen needs to be further optimized. Moreover, the results of the study encourage the initiation of controlled trials to compare the efficacy of DSG with established drugs such as MMF, which will answer the question of the true efficacy of this new drug in therapy of LN. Finally, due to its special mode of action, DSG might qualify as a partner for immunosuppressive combination therapy.

## Abbreviations

ACE: angiotensin converting enzyme; AE: adverse event; AZA: azathioprine; CR: complete response; CRF: case report form; CSA: Cyclosporine A; CTC: common toxicity criteria; CRP: C-reactive protein; CYC: cyclophosphamide; DSG: deoxyspergualin (common name of Gusperimus: DSG); EGFR: estimated glomerular filtration rate; GN: glomerulonephritis; HCQ: hydrocychloroquine; hsc: heat shock protein c; ITT: intention-to-treat analysis; IV: intravenous (ly); LN: lupus nephritis; MMF: mycophenolate mofetil; n.a.: not available; NF-κB: nuclear factor - κB; OCS: oral corticosteroid(s); PP: per protocol; PR: partial response; SAE: serious adverse event; SC: subcutaneous(ly); SD: stable disease; Selena: Safety of Estrogens in Lupus Erythematosus - National Assessment; SLE: systemic lupus erythematosus; SLEDAI: SLE disease activity index; SOC: system organ class; TF: treatment failure; WBC: white blood cell(s); WHO: World Health Organization.

## Competing interests

This study was supported by Euro Nippon Kayaku, Frankfurt, Germany

PH and KN were employees of Euro Nippon Kayaku. HW received an honorarium for data monitoring from Euro Nippon Kayaku. All other authors declare they have no competing interests.

## Authors' contributions

HML, KN and PAH were responsible for the design and the protocol of the study and the interpretation of the data. WHS, VT, UML, IT, IAH, FH and TA were responsible for the recruitment of the patients for the study and medical care during and after the study, as well as the interpretation of the data. HW (monitor) was responsible for the collection, assembly, on-site control and interpretation of the data.
